# Social isolation, regardless of living alone, is associated with mortality: the Otassha study

**DOI:** 10.3389/fpubh.2024.1365943

**Published:** 2024-03-15

**Authors:** Keigo Imamura, Hisashi Kawai, Manami Ejiri, Hiroyuki Sasai, Hirohiko Hirano, Yoshinori Fujiwara, Kazushige Ihara, Shuichi Obuchi

**Affiliations:** ^1^Tokyo Metropolitan Institute for Geriatrics and Gerontology, Tokyo, Japan; ^2^Faculty of Medicine, Hirosaki University, Aomori, Japan

**Keywords:** social isolation, living alone, interactions with others, prognosis, older adults

## Abstract

**Introduction:**

Social isolation has been recognized as a contributing factor to negative health outcomes. Although living alone is associated with health-related outcomes, existing findings are inconsistent. It is not the act of living alone that may predict poor health, but rather social isolation that can lead to increased mortality risk. This study investigated the combined associations of social isolation and living alone with mortality among community-dwelling older adults.

**Methods:**

We included older adults from Itabashi ward, Tokyo, who participated in comprehensive health checkups. Participants were categorized into four groups based on their social isolation status and living alone. The primary outcome was all-cause mortality, analyzed using Cox proportional hazards models.

**Results:**

Of the 1,106 participants (mean age 73, 42% male), 4.5% experienced both social isolation and living alone. This combination was associated with a worse prognosis regarding all-cause mortality (hazard ratio (HR): 2.08 [95% confidence interval (CI), 1.08–4. 00]). Those who were socially isolated but not living alone also showed a trend towards higher mortality risk (HR: 1.41 [95% CI, 0.90–2.20]). Contrastingly, those who were not socially isolated and lived alone did not show an increased mortality risk (HR: 0.81 [95% CI, 0.44–1.49]).

**Discussion and conclusion:**

Living alone is not inherently associated with a poor prognosis in older adults; however, social isolation was associated with a higher mortality risk. Healthcare providers should focus on enhancing social interactions and support for older adults because of their effects on health rather than solely addressing living arrangements to prevent adverse health events.

## 1 Introduction

Social isolation is characterized by a lack of contact with family, friends, or others (1) and is estimated to affect approximately 25% of older adults (2). The prevalence of social isolation has risen, partly because of the COVID-19 pandemic in 2020, which significantly cut down on in-person interactions (3). This condition is associated with adverse health outcomes, including dementia, cardiovascular disease, and increased mortality (4–6). Therefore, preventing these negative health outcomes in socially isolated older adults is a critical public health concern.

Social isolation is typically defined by objective interactions with others; some past research have incorporated living alone into their assessment of social isolation (7, 8). In recent years, there has been an increase in the number of older adults living alone in communities, and some studies have observed an association between living alone and a higher rate of mortality and dementia (9, 10). Consequently, preventing negative outcomes among older adults living alone has become an important concern. However, there is an inconsistent agreement on the association between living alone and health-related outcomes. Sarwari et al. found that older adults living alone often function better than those living with others because they are required to carry out daily activities on their own (11). Similarly, Zhao et al. found no correlation between living alone and a higher mortality risk (12). Our previous research also indicates that poor social networks, rather than living alone, are associated with adverse events (13).

These findings indicate that living alone does not automatically lead to worse health outcomes; the social isolation that often accompanies it may raise the risk of mortality. However, the specific association between mortality and the combination of social isolation and living alone has not been thoroughly investigated. Understanding these relationships is crucial for preventing adverse health effects in older adults who live alone. In recent years, the frequency of interaction with others, used as an indicator of social isolation, has been segregated between face-to-face and non-face-to-face interactions, and its association with mortality has been reported (7). The importance of non-face-to-face interactions is recognized, as the COVID-19 pandemic has considerably limited face-to-face interactions (14, 15). It is thus essential to differentiate between these types of social isolation and assess how they influence health outcomes when combined with living alone.

This study aimed to examine how the combination of living alone and social isolation affects mortality rates among community-dwelling older adults. Furthermore, we also aimed to assess social isolation considering both face-to-face and non-face-to-face interactions and examine the relationship between the combination of living alone and mortality.

## 2 Materials and methods

### 2.1 Participants

Data were collected from the Otassha Study, an ongoing longitudinal study focused on comprehensive health checkups among community-dwelling older adults. The Otassha Study began in October 2011 and involves annual health checkups to date. At the beginning of the study, we sent a mail recruitment letter to all residents aged 65–84 years who were registered in the Basic Resident Register, excluding institutionalized residents and participants from previous surveys conducted by our institute. We followed up with our participants annually, and new participants were recruited on an annual basis as they turned 65 years of age. These checkups within the cohort were discussed in prior studies (16, 17). For this study, we included participants who participated in either the 2012 or 2013 surveys and who completed evaluations regarding living alone and social isolation. The study adhered to the ethical guidelines of the Declaration of Helsinki. All participants provided written informed consent, and the Tokyo Metropolitan Institute for Geriatrics and Gerontology approved the research protocol (2012-H35).

### 2.2 Social isolation and living alone

Social isolation was defined by the frequency of interactions with others. The study questionnaire has been used to measure social isolation in a number of studies and has been reported to be associated with outcomes like disability and mortality (18, 19). The study participants reported how often they interacted with family not living with them, relatives, friends, and neighbors, both in person and through other means such as by phone, e-mail, or letters. Their answers were classified as follows: (1) 6 or 7 times a week (almost every day), (2) 4 or 5 times a week, (3) 2 or 3 times a week, (4) about once a week, (5) 2 or 3 times a month, (6) about once a month, (7) less than once a month, (8) no contact, or (9) none. Drawing from prior studies, we defined “social isolation as a low frequency of face-to-face interaction” for those with in-person contact less than once a week (18, 19). A similar definition was applied for “social isolation as a low frequency of non-face-to-face interaction.” Social isolation was ultimately defined as having both types of interactions less than once a week.

To ascertain their living status, the participants were asked about their living arrangements. Those who responded with “living alone (not living with others),” were categorized as living alone, while those who answered, “living together,” were placed in the not living alone group.

### 2.3 Other variables

During the checkups, we assessed age, sex, body mass index (BMI) (< 18.5, 18.5–24.9, or ≥ 25 kg/m^2^), self-rated health (SRH), instrumental activities of daily living (IADL), number of comorbidities, alcohol consumption, smoking status (current, former, or never), depressive symptoms, subjective economic status, education years, usual gait speed, and cognitive function. SRH was assessed using a four-point Likert scale: (1) excellent, (2) good, (3) fair, and (4) poor, with responses later categorized as either good (excellent/good) or poor (fair/poor) (20). IADL was evaluated with a subscale from the Tokyo Metropolitan Institute of Gerontology Index of Competence (21), where scores range from 0–5, with 5 indicating full independence. Chronic diseases such as hypertension, stroke, heart disease, diabetes, hyperlipidemia, anemia, kidney disease, chronic obstructive pulmonary disease, and cancer were identified through nurse interviews. According to the number of chronic diseases, the participants were classified into three categories (0, 1, and 2+). Depressive symptoms were assessed using the Zung Self-Rating Depression Scale comprising 20 questions, and a score of 50 or higher was defined as having depressive symptoms (22). Subjective economic status was determined based on the question, “Generally speaking, are you financially comfortable?” This was determined based on the question, “In general, are you financially comfortable?” The participants were asked to answer using one of the following responses: (1) have much financial leeway, (2) have financial leeway, (3) average, (4) financially tight, or (5) financially very tight. If the respondents answered (4) financially tight, or (5) financially very tight, they were classified as having a poor economic status. Usual gait speed was tested over a 5 m course, with an additional 3 m before and after acceleration and deceleration, timed manually with a stopwatch. This speed was recorded once and calculated by dividing the distance by the time (m/s). Individuals with a normal gait speed of <1.0 m/s were classified as having a slow gait speed. Cognitive function was measured using the Mini-Mental State Examination (MMSE), which scores from 0 to 30, with higher scores reflecting better cognitive function. Participants with scores ≤23 were considered to have cognitive impairment (23). The trained examiners administered both gait speed and cognitive function tests.

### 2.4 All-cause mortality

All-cause mortality data were sourced from a database managed by the ward office. This mortality information was provided through the notification of death forms for residents. The follow-up period began on the date of initial participation in the 2012 survey (September 25–October 5, 2012) or the 2013 survey (October 7–18, 2013), which served as the baseline. The follow-up was extended up to November 1, 2020, marking the maximum follow-up duration.

### 2.5 Statistical analysis

To investigate the combined association of social isolation and living alone, participants were categorized into four groups based on these criteria: Group 1, no social isolation and not living alone; Group 2, no social isolation, living alone; Group 3, social isolation, not living alone; and Group 4, social isolation and living alone. We examined the baseline differences between the four groups using *χ*^2^ tests for categorical data and analysis of variance (ANOVA) for continuous data.

We visualized the cumulative survival curves for all-cause mortality across the four groups using the Kaplan–Meier method. Furthermore, we applied the log-rank test to assess survival differences among the groups. The association between the groups and all-cause mortality was examined using Cox proportional hazards regression. This analysis included a univariable model and two multivariable models. Model 1 adjusted for age, sex, BMI, self-rated health, number of comorbidities, and IADL, while model 2 further adjusted for depressive symptoms, subjective economic status, education years, slow gait speed, and cognitive impairment, in addition to the covariates in the first model. We also explored the relationship between social isolation in face-to-face and non-face-to-face interactions, living alone, and mortality using the same Cox proportional hazards model. In sensitivity analyses of the possible influence of reverse causation, we performed sensitivity analyses using the same statistical approach, after excluding individuals who died during the first 1 year of follow-up. Furthermore, to examine whether the association between the combination of social isolation and living alone and mortality differed by age and sex, we performed subgroup analyses by stratifying the sample into two age groups (65–74 and 75+) and by sex.

Descriptive results are shown using the data, including missing values. For missing data on confounders, we performed multiple imputations using the chained equations method, assuming that the analyzed data were missing at random (24). Results from 20 imputed data sets were combined for analysis using Rubin’s formula. The following variables were incorporated into the imputation model: social isolation, living arrangement, covariates, and outcome variables.

## 3 Results

### 3.1 Participant characteristics

Out of the total 1,122 participants surveyed in 2012–2013, 16 were excluded because of missing data on social isolation or living arrangements, leaving 1,106 participants for the final analysis. Table 1 presents the participant characteristics, stratified by combined social isolation and living alone. Of the total sample, 532 participants (48.1%) experienced a low frequency of face-to-face interaction, while 394 (35.6%) had a low frequency of non-face-to-face interaction. A total of 243 participants (22.0%) were experiencing social isolation, and 260 (23.5%) were living alone. Additionally, 50 participants (4.5%) were identified as group 4, experiencing both social isolation and living alone. The *χ*^2^ tests and ANOVA revealed that individuals in group 4 were typically older, male, with poorer self-rated health, less likely to have never consumed alcohol or smoked, had more depressive symptoms and poor subjective financial status, shorter education years, had slower gait speeds, and had lower MMSE scores. Group 3 was the only group in which fewer than 90% of participants were independent in terms of IADLs.

**Table 1:** Characteristics of participants stratified by the combinations of social isolation and living alone.

	Missing	Total	No SI × Not LA	No SI × LA	SI × Not LA	SI × LA	*p*-value	*N* = 1,106	*N* = 653	*N* = 210	*N* = 193	*N* = 50
Age (years)	0	73.0 (5.6)	72.7 (5.5)	73.1 (5.8)	73.6 (5.7)	74.2 (6.2)	0.07
Male (%)	0	41.5	42.9	18.1	57.0	62.0	<0.01
BMI (kg/m^2^)	8	23.0 (3.4)	23.1 (3.5)	22.9 (3.4)	22.5 (2.8)	22.7 (3.4)	0.16
<18.5		7.5	7.3	7.2	7.3	12.0	0.24
18.5–24.9		67.8	66.2	67.5	74.9	62.0	
≥ 25.0		24.8	26.5	25.4	17.8	26.0	
Self-rated health, poor (%)	36	18.3	15.2	19.8	22.2	36.7	<0.01
Number of chronic diseases (%)	3						0.68
0		31.2	30.5	33.8	33.2	22.0	
1		33.7	33.5	34.8	32.1	38.0	
2+		35.1	36.0	31.4	34.7	40.0	
IADL, full mark (%)	23	91.9	92.7	96.6	83.2	93.8	<0.01
Alcohol consumption status (%)	1						<0.01
Current		52.3	54.2	43.1	57.0	48.0	
Former		8.5	5.4	10.0	14.0	22.0	
Never		39.2	40.4	46.9	29.0	30.0	
Smoking status (%)	1						<0.01
Current		11.8	10.1	10.5	15.0	26.0	
Former		26.0	25.4	15.8	35.8	38.0	
Never		62.3	64.5	73.7	49.2	36.0	
Zung Depression Scale	12	34.6	33.5	35.8	35.3	40.7	<0.01
Depressive symptoms (%)		6.5	4.5	8.7	7.5	20.0	<0.01
Poor subjective financial status (%)	9	21.9	19.9	20.1	26.2	38.0	<0.01
Education years (years)	9	12.4 (2.7)	12.7 (2.7)	12.2 (2.7)	12.4 (2.7)	11.0 (2.5)	<0.01
Usual gait speed (m/s)	20	1.3 (0.3)	1.4 (0.2)	1.3 (0.3)	1.3 (0.3)	1.2 (0.3)	<0.01
<1.0 m/s		8.4	6.4	9.2	12.1	16.7	0.01
MMSE score	26	28.1 (2.3)	28.2 (2.2)	28.2 (2.1)	27.6 (2.6)	27.7 (1.9)	0.02
≤ 23 points		4.5	4.2	3.9	6.4	4.2	0.61

Data are presented as mean (SD) for continuous measures, and *n* (%) for categorical measures.

BMI, body mass index; IADL, instrumental activity of daily living; MMSE, mini-mental state examination; SI, social isolation; LA, living alone.

### 3.2 Association between combined social isolation and living alone and all-cause mortality

**Figure 1:**
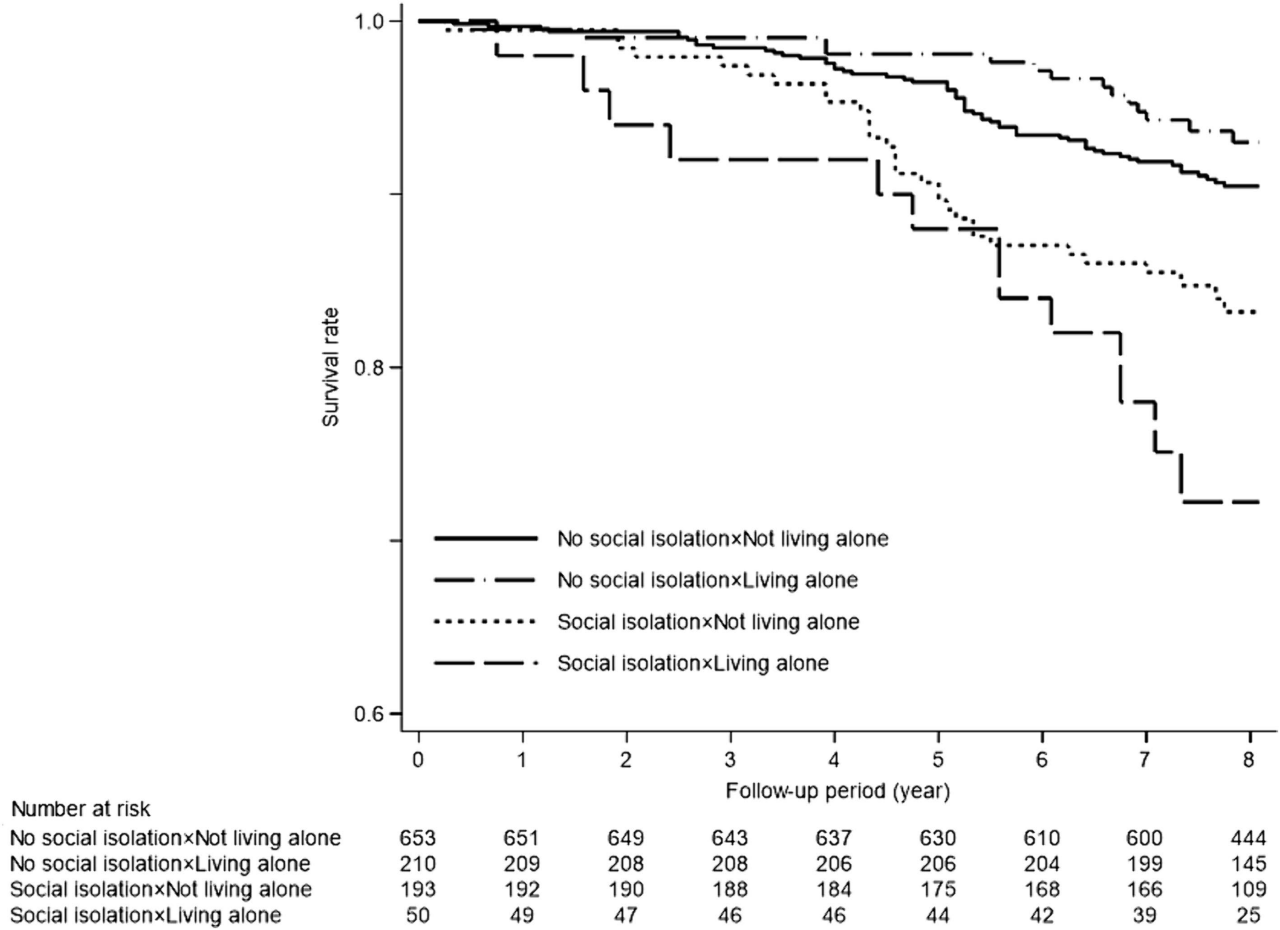
Results of Kaplan–Meier analyses for the incidence of death.

During the median follow-up period of 96 months (interquartile range: 84–97 months), a total of 118 deaths occurred. Figure 1 presents the Kaplan–Meier survival analysis results for the four groups categorized by social isolation and living alone. The log-rank test indicated that groups 3 and 4 experienced significantly lower survival rates (*p* < 0.001).

Table 2 shows the association between the combination of social isolation and living alone and all-cause mortality. In model 2, group 4 had a significantly higher risk of mortality (hazard ratio (HR): 2.08 [95% confidence interval (CI), 1.08–4.00]). Group 3 did not show a statistically significant association with increased mortality risk (HR: 1.41 [95% CI, 0.90–2.20]), but had a higher incidence rate (IR) than groups 1 and 2 (group 1, IR: 12.2, group 2, IR: 8.7, group 3, IR: 22.3). The combination of social isolation based on low frequency of face-to-face interactions and living alone did not show a statistically significant association in any group; however, the group of social isolation (group 3, IR: 16.1, group 4, IR: 22.7) had higher IR than the no social isolation group (group 1, IR: 12.8, group 2, IR: 7.2). The combination of social isolation based on low frequency of non-face-to-face interactions and living alone showed a significantly higher risk of mortality in the social isolation group regardless of living alone status (group 3, HR: 1.63 [95% CI, 1.07–2.48], group 4, HR: 1.98 [95% CI, 1.04–3.77]).

**Table 2:** Association between combination of social isolation and living alone and all-cause mortality.

	Number of events	IR*	Crude model		Adjusted model 1		Adjusted model 2		HR (95% CI)	*p* value	HR (95% CI)	*p* value	HR (95% CI)	*p* value
Social isolation × living alone								
No social isolation × Not living alone	60	12.2	Reference		Reference		Reference	
No social isolation × Living alone	14	8.7	0.71 (0.40–1.27)	0.25	0.86 (0.47–1.57)	0.62	0.81 (0.44–1.49)	0.50
Social isolation × Not living alone	31	22.3	1.86 (1.20–2.87)	<0.01	1.47 (0.94–2.29)	0.08	1.41 (0.90–2.20)	0.13
Social isolation × Living alone	13	37.6	3.14 (1.72–5.72)	<0.01	2.47 (1.33–4.60)	<0.01	2.08 (1.08–4.00)	0.03
Social isolation (defined as Low frequency of face to face contact) × living alone								
No social isolation × Not living alone	41	12.8	Reference		Reference		Reference	
No social isolation × Living alone	8	7.2	0.56 (0.26–1.19)	0.13	0.66 (0.30–1.42)	0.28	0.62 (0.28–1.35)	0.23
Social isolation × Not living alone	50	16.1	1.26 (0.84–1.91)	0.27	1.06 (0.70–1.61)	0.78	1.04 (0.69–1.59)	0.84
Social isolation × Living alone	19	22.7	1.78 (1.04–3.07)	0.04	1.77 (1.02–3.09)	0.04	1.53 (0.86–2.72)	0.15
Social isolation (defined as Low frequency of non-face to face contact) × living alone								
No social isolation × Not living alone	42	10.6	Reference		Reference		Reference	
No social isolation × Living alone	13	9.1	0.86 (0.46–1.61)	0.64	1.01 (0.53–1.93)	0.98	0.95 (0.50–1.82)	0.89
Social isolation × Not living alone	49	21.1	2.03 (1.34–3.06)	<0.01	1.67 (1.10–2.54)	0.02	1.63 (1.07–2.48)	0.02
Social isolation × Living alone	14	26.7	2.56 (1.40–4.68)	<0.01	2.31 (1.24–4.28)	0.01	1.98 (1.04–3.77)	0.03

IR, incidence rate; CI, confidence interval; HR, hazard ratio; SI, social isolation; LA, living alone; BMI, body mass index; IADL, instrumental activity of daily living; MMSE, mini-mental state examination. Adjusted model 1: age; sex, BMI, self-rated health, number of comorbidities, and IADL. Adjusted model 2: Adjusted model 1+ depressive symptoms, subjective financial status, education years, slow gait speed, and cognitive impairment. Incidence rate* = incidence rate per 1,000 person-years.

In the analyses that excluded mortality that occurred during the first 1 year, although the associations of combined social isolation and living alone with outcomes were weakened, the results were not substantially different from those of the primary analyses (Supplementary Table S1). In the subgroup analyses by sex and age group, the association between combined social isolation and living alone and mortality was attenuated in some analyses in groups 3 and 4, but as in the primary analysis, the group of social isolation had a higher IR regardless of living alone. *p*-values for tests of interaction were not significant (Supplementary Tables S2, S3).

## 4 Discussion

These results showed that the co-existence of social isolation and living alone was associated with a worse prognosis. Specifically, a significant reduction in non-face-to-face interactions was associated with a poorer prognosis, independent of living alone. These results suggest that living alone does not necessarily increase the risk of mortality, but social isolation, characterized by a decrease in interactions, is associated with an increased mortality risk.

In this study, group 4 (social isolation and living alone) was independently associated with worse prognosis, even after adjusting for subject characteristics, psychological and social factors, cognitive function, and gait speed. Group 3 (social isolation and not living alone) also had a higher IR of mortality, although this was not statistically significant. That is, social isolation was associated with higher risk of mortality, regardless of whether the participants lived alone. Although previous studies have found an association between social isolation or living alone and prognosis separately (6, 9), this is the first study to report the combined effect of social isolation and living alone on prognosis. Several mechanisms have been reported to be associated with social isolation and mortality (25). These include physiological factors, such as activation of the hypothalamic–pituitary–adrenal axis and increased chronic stress response; psychological factors, such as more likely to be depressed or suicidal; and behavioral factors, like more likely to smoke, drink alcohol, and make poor dietary choices and no exercise habit (25). In the present data, the socially isolated groups (groups 3 and 4) were characterized by a higher percentage of people with poor self-rated health and depressive symptoms, and a higher percentage of people with a history of drinking and smoking. Thus, these factors may have triggered physiological factors such as activation of the hypothalamic–pituitary–adrenal axis and increased chronic stress response, which may have been associated with poor prognosis. However, since this study was could not investigate physiological factors, these mechanisms need to be further verified.

Conversely, group 2 (not socially isolated x living alone) showed no association with mortality. A previous study also reported that older adults living alone was not associated with mortality (12), which is consistent with the present findings. Older adults living alone, especially women, may be physically healthier than older adults living with others (11), and furthermore, older adults who interact with others, as in group 2, may receive more encouragement from those around them to maintain a healthy lifestyle (26, 27). These findings underscore the importance of assessing social isolation, rather than living arrangements, when determining the risk of adverse events in older adults.

We categorized the frequency of interaction as face-to-face or non-face-to-face and examined its association with reduced interaction, living alone, and all-cause mortality. Results showed that regardless of whether a participant lived alone or not, reduced non-face-to-face interactions, such as phone calls and e-mails, were associated with worse prognosis. Recent studies have reported that non-face-to-face interactions have protective effects on health outcomes (28, 29). Non-face-to-face interactions make it easier to seek advice, exchange health information, and receive support from distant relatives and friends. These benefits may be important not only for the older adults themselves, but also for distant family and friends to help prevent health problems in the older adults. However, reduced face-to-face interaction was not significantly associated with mortality. Previous studies have found that face-to-face interaction is also effective in preventing disability and maintaining mental health (28, 30). A difference with the results of the present study is the possible confounding of depressive symptoms, gait ability, and cognitive function. In the present study, the association with mortality was not significant after adjustment for variables such as depressive symptoms, slow gait speed, and cognitive decline, so the reduction in face-to-face interactions may be more critical in older adults with depressive symptoms, slow gait speed, or cognitive decline. However, the small number of such subjects in the overall study population may have masked these effects. Future studies are needed in populations with a wide range of subject characteristics, such as frailty and mild cognitive impairment.

This study has several limitations. First, this study was conducted in Itabashi ward, Tokyo, which is an urban area. Therefore, there may be differences in characteristics between older adults living in this area and those living in rural areas. Moreover, the participants of this study were not randomly selected, which may introduce a selection bias toward older adults with high health awareness. In fact, most of the participants of this study had high IADL ability (>90% independent) and normal gait speed, indicating a high level of functioning and mobility. Previous research has shown a higher risk of adverse health outcomes in older adults with frailty and those living alone with reduced functional capacity (11). Therefore, we should be cautious when applying our findings to frail individuals or older adults living alone with reduced functional capacity, as they may have different patterns and consequences of social isolation and living alone. Second, we measured non-face-to-face interactions through phone calls and e-mails but did not account for the use of social media, which has become increasingly popular among older adults with the rise of communication technology (31). Third, there may also be unmeasured confounders that may affect both social isolation and mortality, such as marital history, reason for living alone, onset and duration of social isolation and living alone. Therefore, we cannot rule out the possibility of residual confounding by other factors that we did not measure or control for. This information would allow a more detailed analysis of the association between the combination of social isolation and living alone and mortality. Finally, in this study, we defined social isolation based on the frequency of interactions with others. This method has been widely used in previous studies targeting community-dwelling older adults. However, there is no standardized method for measuring social isolation, and some studies have evaluated social isolation from the aspects of social support and social activities (32). Therefore, this study may have only assessed one aspect of social isolation. Future studies may need to evaluate social isolation from a broader perspective of social relationships.

In conclusion, this study has shown that social isolation is associated with a poor prognosis, regardless of whether one lives alone. Older adults living alone do not necessarily have a higher risk for health-related outcomes, so the frequency of interactions with others and support from others should also be assessed. Health care providers should focus on assessing the social isolation status of older adults to prevent adverse events.

## Data availability statement

The data analyzed in this study is subject to the following licenses/restrictions: the datasets presented in this article are not readily available because of ethical and privacy restrictions. Requests to access these datasets should be directed to imamura@tmig.or.jp.

## Ethics statement

The studies involving humans were approved by Ethics committee of the Tokyo Metropolitan Institute for Geriatrics and Gerontology. The studies were conducted in accordance with the local legislation and institutional requirements. The participants provided their written informed consent to participate in this study.

## Author contributions

KIm: Formal analysis, Methodology, Software, Visualization, Writing – original draft. HK: Data curation, Investigation, Methodology, Project administration, Resources, Validation, Writing –review & editing. ME: Investigation, Writing –review & editing. HS: Investigation, Resources, Writing – review & editing. HH: Investigation, Resources, Writing – review & editing. YF: Investigation, Resources, Writing – review & editing. KIh: Investigation, Resources, Writing – review & editing. SO: Conceptualization, Data curation, Funding acquisition, Project administration, Resources, Supervision, Writing – review & editing.
