# Supplementary material for: Social isolation, regardless of living alone, is associated with mortality: the Otassha study
Source: Front Public Health. 2024 Mar 15;12:1365943. doi: 10.3389/fpubh.2024.1365943 (PMC10978664; doi:10.3389/fpubh.2024.1365943)
Supplement: Supplementary file 1 [file Data_Sheet_1.docx]

| Supplemental table 1. Association between combination of social isolation and living alone and all-cause mortality after excluding deaths within 1 year from the baseline (n=1101) | | | | | | | | | | |  |
| --- | --- | --- | --- | --- | --- | --- | --- | --- | --- | --- | --- |
|  |  | Crude model | | | Adjusted model 1 | | | Adjusted model 2 | | |  |
|  | Number of events | IR* | HR  (95％CI) | P value | | HR  (95％CI) | P value | | HR  (95％CI) | P value |  |
| Social isolation × living alone | | | | | |  |  | |  |  |  |
| No social isolation × Not living alone | 58 | 11.8 | reference |  | |  |  | |  |  |  |
| No social isolation × Living alone | 13 | 8.1 | 0.68  (0.37-1.25) | 0.21 | | 0.84  (0.45-1.58) | 0.60 | | 0.80  (0.43-1.50) | 0.49 |  |
| Social isolation × Not living alone | 30 | 21.6 | 1.86  (1.20-2.90) | 0.01 | | 1.46  (0.93-2.30) | 0.10 | | 1.41  (0.89-2.22) | 0.14 |  |
| Social isolation × Living alone | 12 | 34.7 | 3.01  (1.62-5.61) | <0.01 | | 2.42  (1.27-4.60) | 0.01 | | 2.06  (1.05-4.04) | 0.04 |  |
| Low frequency of face to face contact × living alone | | | | | |  |  | |  |  |  |
| No social isolation × Not living alone | 40 | 12.5 | reference |  | |  |  | |  |  |  |
| No social isolation × Living alone | 7 | 6.3 | 0.50  (0.22-1.11) | 0.09 | | 0.60  (0.27-1.37) | 0.23 | | 0.57  (0.25-1.30) | 0.18 |  |
| Social isolation × Not living alone | 48 | 15.5 | 1.24  (0.82-1.89) | 0.31 | | 1.04  (0.68-1.59) | 0.85 | | 1.03  (0.67-1.57) | 0.91 |  |
| Social isolation × Living alone | 18 | 21.5 | 1.73  (0.99-3.03) | 0.05 | | 1.76  (1.00-3.11) | 0.05 | | 1.54  (0.86-2.77) | 0.15 |  |
| Low frequency of non-face to face contact × living alone | | | | | |  |  | |  |  |  |
| No social isolation × Not living alone | 40 | 10.1 | reference |  | |  |  | |  |  |  |
| No social isolation × Living alone | 12 | 8.4 | 0.84  (0.44-1.59) | 0.59 | | 1.01  (0.51-1.97) | 0.99 | | 0.96  (0.49-1.87) | 0.90 |  |
| Social isolation × Not living alone | 48 | 20.6 | 2.09  (1.38-3.18) | <0.01 | | 1.72  (1.12-2.64) | 0.01 | | 1.68  (1.09-2.59) | 0.02 |  |
| Social isolation × Living alone | 13 | 24.8 | 2.50  (1.34-4.67) | <0.01 | | 2.29  (1.21-4.35) | 0.01 | | 1.99  (1.03-3.87) | 0.04 |  |
| IR : incidence rate, CI : confidence interval, HR : hazard ratio, SI : social isolation, LA : living alone, BMI: body mass index, IADL: instrumental activity of daily living, MMSE: mini-mental state examination.  Adjusted model 1 : age, sex, BMI, self-rated health, number of comorbidities, and IADL.  Adjusted model 2 : Adjusted model 1+ depressive symptoms, subjective financial status, education years, slow gait speed, and cognitive impairment.  Incidence rate* = incidence rate per 1000 person-years. | | | | | | | | | | | |

| Supplemental table 2. Association between combination of social isolation and living alone and all-cause mortality by age group. | | | | | | | | | | | | |  |
| --- | --- | --- | --- | --- | --- | --- | --- | --- | --- | --- | --- | --- | --- |
|  |  |  |  | | Crude model | | | Adjusted model 1 | | Adjusted model 2 | |  |  |
|  |  | Number of events | | IR* | | HR  (95％CI) | P value | HR  (95％CI) | P value | HR  (95％CI) | P value | P for interaction |  |
| 65-74 | Social isolation × living alone | | | | | | |  |  |  |  | 0.46 |  |
| (n=669) | No social isolation × Not living alone | 17 | | 9.6 | | reference |  |  |  |  |  |  |  |
|  | No social isolation × Living alone | 5 | | 7.5 | | 0.99  (0.36-2.67) | 0.98 | 1.11  (0.41-3.02) | 0.84 | 1.04  (0.37-2.88) | 0.95 |  |  |
|  | Social isolation × Not living alone | 11 | | 18.2 | | 2.61  (1.22-5.58) | 0.01 | 2.18  (1.00-4.75) | 0.049 | 2.15  (0.96-4.80) | 0.06 |  |  |
|  | Social isolation × Living alone | 4 | | 31.6 | | 3.38  (1.14-10.03) | 0.3 | 2.37  (0.78-7.20) | 0.13 | 2.01  (0.61-6.60) | 0.25 |  |  |
|  | Low frequency of face to face contact × living alone | | | | | | |  |  |  |  | 0.63 |  |
|  | No social isolation × Not living alone | 11 | | 9.8 | | reference |  |  |  |  |  |  |  |
|  | No social isolation × Living alone | 4 | | 8.3 | | 1.20  (0.38-3.77) | 0.76 | 1.34  (0.42-4.26) | 0.62 | 1.27  (0.39-4.11) | 0.69 |  |  |
|  | Social isolation × Not living alone | 17 | | 13.6 | | 1.73  (0.81-3.70) | 0.16 | 1.31  (0.60-2.85) | 0.49 | 1.29  (0.59-2.83) | 0.52 |  |  |
|  | Social isolation × Living alone | 5 | | 16.2 | | 1.78  (0.62-5.13) | 0.28 | 1.36  (0.46-3.97) | 0.58 | 1.10  (0.36-3.40) | 0.87 |  |  |
|  | Low frequency of non-face to face contact × living alone | | | | | | |  |  |  |  | 0.31 |  |
|  | No social isolation × Not living alone | 11 | | 7.9 | | reference |  |  |  |  |  |  |  |
|  | No social isolation × Living alone | 5 | | 8.1 | | 1.46  (0.51-4.21) | 0.48 | 1.59  (0.55-4.62) | 0.39 | 1.53  (0.52-4.49) | 0.44 |  |  |
|  | Social isolation × Not living alone | 17 | | 17.3 | | 3.00  (1.40-6.40) | 0.01 | 2.61  (1.21-5.64) | 0.01 | 2.62  (1.17-5.83) | 0.02 |  |  |
|  | Social isolation × Living alone | 4 | | 23.2 | | 2.67  (0.85-8.39) | 0.09 | 2.23  (0.70-7.14) | 0.18 | 1.88  (0.53-6.58) | 0.33 |  |  |
| ≥ 75 | Social isolation × living alone |  | |  | |  |  |  |  |  |  |  |  |
| (n=437) | No social isolation × Not living alone | 43 | | 24.3 | | reference |  |  |  |  |  |  |  |
|  | No social isolation × Living alone | 9 | | 13.6 | | 0.55  (0.27-1.12) | 0.10 | 0.80  (0.37-1.71) | 0.56 | 0.75  (0.35-1.61) | 0.45 |  |  |
|  | Social isolation × Not living alone | 20 | | 33.0 | | 1.39  (0.82-2.36) | 0.22 | 1.34  (0.77-2.31) | 0.30 | 1.21  (0.69-2.13) | 0.50 |  |  |
|  | Social isolation × Living alone | 9 | | 71.1 | | 3.10  (1.51-6.37) | <0.01 | 3.27  (1.52-7.04) | 0.01 | 2.71  (1.20-6.12) | 0.02 |  |  |
|  | Low frequency of face to face contact × living alone | | | | | | |  |  |  |  |  |  |
|  | No social isolation × Not living alone | 30 | | 26.6 | | reference |  |  |  |  |  |  |  |
|  | No social isolation × Living alone | 4 | | 8.3 | | 0.30  (0.11-0.86) | 0.03 | 0.46  (0.16-1.35) | 0.16 | 0.42  (0.14-1.24) | 0.12 |  |  |
|  | Social isolation × Not living alone | 33 | | 26.5 | | 1.00  (0.61-1.63) | 0.99 | 0.95  (0.58-1.56) | 0.83 | 0.87  (0.52-1.45) | 0.59 |  |  |
|  | Social isolation × Living alone | 14 | | 45.3 | | 1.74  (0.92-3.28) | 0.09 | 2.14  (1.10-4.17) | 0.02 | 1.79  (0.90-3.54) | 0.10 |  |  |
|  | Low frequency of non-face to face contact × living alone | | | | | | |  |  |  |  |  |  |
|  | No social isolation × Not living alone | 31 | | 22.3 | | reference |  |  |  |  |  |  |  |
|  | No social isolation × Living alone | 8 | | 12.9 | | 0.57  (0.26-1.25) | 0.16 | 0.84  (0.37-1.90) | 0.67 | 0.79  (0.35-1.81) | 0.58 |  |  |
|  | Social isolation × Not living alone | 32 | | 32.6 | | 1.50  (0.92-2.46) | 0.11 | 1.46  (0.88-2.42) | 0.14 | 1.41  (0.84-2.36) | 0.19 |  |  |
|  | Social isolation × Living alone | 10 | | 57.9 | | 2.71  (1.33-5.52) | 0.01 | 3.13  (1.48-6.61) | <0.01 | 2.59  (1.18-5.64) | 0.02 |  |  |
| IR : incidence rate, CI : confidence interval, HR : hazard ratio, SI : social isolation, LA : living alone, BMI: body mass index, IADL: instrumental activity of daily living, MMSE: mini-mental state examination.  Adjusted model 1 : sex, BMI, self-rated health, number of comorbidities, and IADL.  Adjusted model 2 : Adjusted model 1+ depressive symptoms, subjective financial status, education years, slow gait speed, and cognitive impairment.  Incidence rate* = incidence rate per 1000 person-years. | | | | | | | | | | | | | |

| Supplemental table 3. Association between combination of social isolation and living alone and all-cause mortality by sex. | | | | | | | | | | | | |  |
| --- | --- | --- | --- | --- | --- | --- | --- | --- | --- | --- | --- | --- | --- |
|  |  |  |  | | Crude model | | | Adjusted model 1 | | Adjusted model 2 | |  |  |
|  |  | Number of events | | IR* | | HR  (95％CI) | P value | HR  (95％CI) | P value | HR  (95％CI) | P value | P for interaction |  |
| Male | Social isolation × living alone | | | | | | |  |  |  |  | 0.42 |  |
| (n=459) | No social isolation × Not living alone | 39 | | 18.9 | | reference |  |  |  |  |  |  |  |
|  | No social isolation × Living alone | 4 | | 14.2 | | 0.75  (0.27-2.10) | 0.59 | 0.84  (0.29-2.42) | 0.75 | 0.76  (0.26-2.21) | 0.61 |  |  |
|  | Social isolation × Not living alone | 23 | | 29.5 | | 1.60  (0.96-2.68) | 0.07 | 1.37  (0.81-2.33) | 0.24 | 1.27  (0.74-2.18) | 0.39 |  |  |
|  | Social isolation × Living alone | 9 | | 43.2 | | 2.37  (1.15-4.89) | 0.02 | 1.95  (0.91-4.20) | 0.09 | 1.52  (0.66-3.50) | 0.32 |  |  |
|  | Low frequency of face to face contact × living alone | | | | | | |  |  |  |  | 0.83 |  |
|  | No social isolation × Not living alone | 24 | | 19.3 | | reference |  |  |  |  |  |  |  |
|  | No social isolation × Living alone | 3 | | 16.2 | | 0.83  (0.25-2.77) | 0.77 | 0.90  (0.26-3.05) | 0.86 | 0.82  (0.23-2.90) | 0.76 |  |  |
|  | Social isolation × Not living alone | 38 | | 23.6 | | 1.23  (0.74-2.05) | 0.43 | 1.18  (0.71-1.98) | 0.52 | 1.11  (0.66-1.87) | 0.69 |  |  |
|  | Social isolation × Living alone | 10 | | 32.7 | | 1.72  (0.82-3.61) | 0.15 | 1.64  (0.76-3.53) | 0.21 | 1.25  (0.54-2.87) | 0.60 |  |  |
|  | Low frequency of non-face to face contact × living alone | | | | | | |  |  |  |  | 0.97 |  |
|  | No social isolation × Not living alone | 26 | | 15.9 | | reference |  |  |  |  |  |  |  |
|  | No social isolation × Living alone | 3 | | 13.1 | | 0.82  (0.25-2.72) | 0.75 | 0.98  (0.29-3.32) | 0.98 | 0.83  (0.24-2.89) | 0.78 |  |  |
|  | Social isolation × Not living alone | 36 | | 29.4 | | 1.90  (1.14-3.14) | 0.01 | 1.79  (1.07-3.00) | 0.03 | 1.70  (1.00-2.87) | 0.05 |  |  |
|  | Social isolation × Living alone | 10 | | 38.2 | | 2.47  (1.19-5.12) | 0.02 | 2.14  (1.00-4.58) | 0.05 | 1.75  (0.78-3.93) | 0.18 |  |  |
| Female | Social isolation × living alone |  | |  | |  |  |  |  |  |  |  |  |
| (n=647) | No social isolation × Not living alone | 21 | | 7.4 | | reference |  |  |  |  |  |  |  |
|  | No social isolation × Living alone | 10 | | 7.6 | | 1.02  (0.48-2.17) | 0.96 | 0.86  (0.40-1.85) | 0.70 | 0.84  (0.39-1.83) | 0.66 |  |  |
|  | Social isolation × Not living alone | 8 | | 13.1 | | 1.79  (0.79-4.04) | 0.16 | 1.67  (0.73-3.83) | 0.22 | 1.70  (0.74-3.95) | 0.21 |  |  |
|  | Social isolation × Living alone | 4 | | 29.1 | | 3.94  (1.35-11.48) | 0.01 | 3.66  (1.24-10.82) | 0.02 | 3.63  (1.18-11.21) | 0.03 |  |  |
|  | Low frequency of face to face contact × living alone | | | | | | |  |  |  |  |  |  |
|  | No social isolation × Not living alone | 17 | | 8.7 | | reference |  |  |  |  |  |  |  |
|  | No social isolation × Living alone | 5 | | 5.4 | | 0.62  (0.23-1.67) | 0.34 | 0.49  (0.18-1.35) | 0.17 | 0.49  (0.18-1.35) | 0.17 |  |  |
|  | Social isolation × Not living alone | 12 | | 8.0 | | 0.93  (0.44-1.94) | 0.84 | 0.83  (0.39-1.75) | 0.62 | 0.85  (0.40-1.80) | 0.67 |  |  |
|  | Social isolation × Living alone | 9 | | 17.0 | | 1.95  (0.87-4.37) | 0.11 | 1.73  (0.76-3.93) | 0.19 | 1.69  (0.73-3.92) | 0.22 |  |  |
|  | Low frequency of non-face to face contact × living alone | | | | | | |  |  |  |  |  |  |
|  | No social isolation × Not living alone | 16 | | 6.8 | | reference |  |  |  |  |  |  |  |
|  | No social isolation × Living alone | 10 | | 8.4 | | 1.22  (0.56-2.70) | 0.62 | 0.92  (0.41-2.06) | 0.83 | 0.90  (0.40-2.03) | 0.80 |  |  |
|  | Social isolation × Not living alone | 13 | | 11.8 | | 1.74  (0.84-3.63) | 0.14 | 1.40  (0.66-2.95) | 0.38 | 1.41  (0.67-2.99) | 0.37 |  |  |
|  | Social isolation × Living alone | 4 | | 15.2 | | 2.23  (0.74-6.66) | 0.15 | 2.50  (0.83-7.55) | 0.10 | 2.36  (0.76-7.40) | 0.14 |  |  |
| IR : incidence rate, CI : confidence interval, HR : hazard ratio, SI : social isolation, LA : living alone, BMI: body mass index, IADL: instrumental activity of daily living, MMSE: mini-mental state examination.  Adjusted model 1 : age, BMI, self-rated health, number of comorbidities, and IADL.  Adjusted model 2 : Adjusted model 1+ depressive symptoms, subjective financial status, education years, slow gait speed, and cognitive impairment.  Incidence rate* = incidence rate per 1000 person-years. | | | | | | | | | | | | | |
